# Radiation meets inflammation: NLRP3 inflammasome at the core of radiation-induced cardiac injury

**DOI:** 10.1186/s12967-025-07377-3

**Published:** 2025-11-21

**Authors:** Caterina Boncompagni, Stefania Giacovazzi, Mariasole Perrone, Sonia Missiroli, Giulio Righes, Paolo Pinton, Carlotta Giorgi

**Affiliations:** 1 Department of Medical Sciences, LTTA Laboratory for Advanced Therapies, University of Ferrara, Technopole of Ferrara, 44121 Ferrara, Italy; 2 Maria Cecilia Hospital, GVM Care & Research, 48033 Cotignola (RA), Italy; 3 Biomedical Research Center, Kansai Medical University, 1-9-11 Shin- machi, Hirakata, Osaka 573-1191 Japan; 4 IRCCS San Camillo Hospital, 30126 Venice, Italy

**Keywords:** NLRP3 inflammasome, Cardiovascular disease, Radiotherapy, Inhibitors

## Abstract

Radiation therapy is a widely used and effective treatment for various types of cancer; however, it is associated with several side effects, including an increased risk of cardiovascular disease. While the molecular mechanisms underlying RT-induced cardiovascular damage remain unclear, the NLRP3 inflammasome has emerged as a central player in its pathogenesis. This review focuses on the current understanding of the involvement of NLRP3 in radiation-induced cardiotoxicity, highlighting emerging evidence reported in the literature. Recent findings shown that the NLRP3 inflammasome may play a role in radiation-induced cardiovascular diseases, drawing attention to it as a potential therapeutic target. We summarize key findings on the role of radiation in modulating the NLRP3 inflammasome and inducing cardiovascular disease, providing a detailed overview of NLRP3 inflammasome functions in different radiation-induced cardiovascular diseases. Moreover, we discuss the therapeutic potential of targeting NLRP3 to prevent radiation-induced cardiovascular diseases. The development of effective treatments for radiotherapy-induced cardiotoxicity is becoming increasingly urgent and understanding NLRP3-dependent mechanisms could pave the way for innovative therapeutic approaches.

## Introduction

Radiation therapy (RT) is a widely used and effective cancer treatment for various types of cancer, including breast cancer, Hodgkin’s lymphoma, thymus cancer, and lung cancer. Its functioning is based on the administration of high-energy ionizing radiation, such as X-rays and gamma rays (γ), which kill cancer cells and shrink tumors. These charged particles deposit their energy in the tissues they pass through, killing cancer cells or inducing genetic mutations that lead to cell death. Although on one hand this treatment has evident benefits in terms of tumor reduction, on the other hand, it leads to several side effects, including the development of cardiovascular diseases. RT potentially causes damage to the pericardium, valves, conduction system, and arteries. The risk of cardiovascular events increases in the years following treatment, with a prevalence ranging from 10% to 30% after 5–10 years [1]. The development of such complications depends on the radiation site, the administered dosage, and the presence of pre-existing cardiovascular risk factors.

The pathophysiological mechanism of radiation-induced cardiovascular diseases (RICVD) develops through direct and indirect pathways. A direct action of radiations causes single-stranded (SSB) and double-stranded (DSB) damage in DNA, leading to the inhibition of cell proliferation [2–4]. An indirect action is mainly determined by water radiolysis which generates reactive oxygen species (ROS) and causes oxidative stress resulting in base modifications and DSBs [3, 5].

Although the molecular mechanism leading to the development of RT-induced cardiovascular diseases is still unknown, the scientific community has focused on the NOD-like receptor family pyrin domain-containing 3 (NLRP3) inflammasome as a potential key player in the onset of this category of diseases. Coherently, the release of cytosolic contents as a consequence of cell death, and ROS production are upstream signals of the NLRP3 inflammasome pathway [6, 7].

In this review, we focus on and discuss the role of the NLRP3 inflammasome in cardiovascular diseases induced by radiotherapy and how its modulation could represent a potential therapeutic target to improve cancer survivors’ quality of life.

### The NLRP3 inflammasome: structure and regulation

**Fig. 1:**
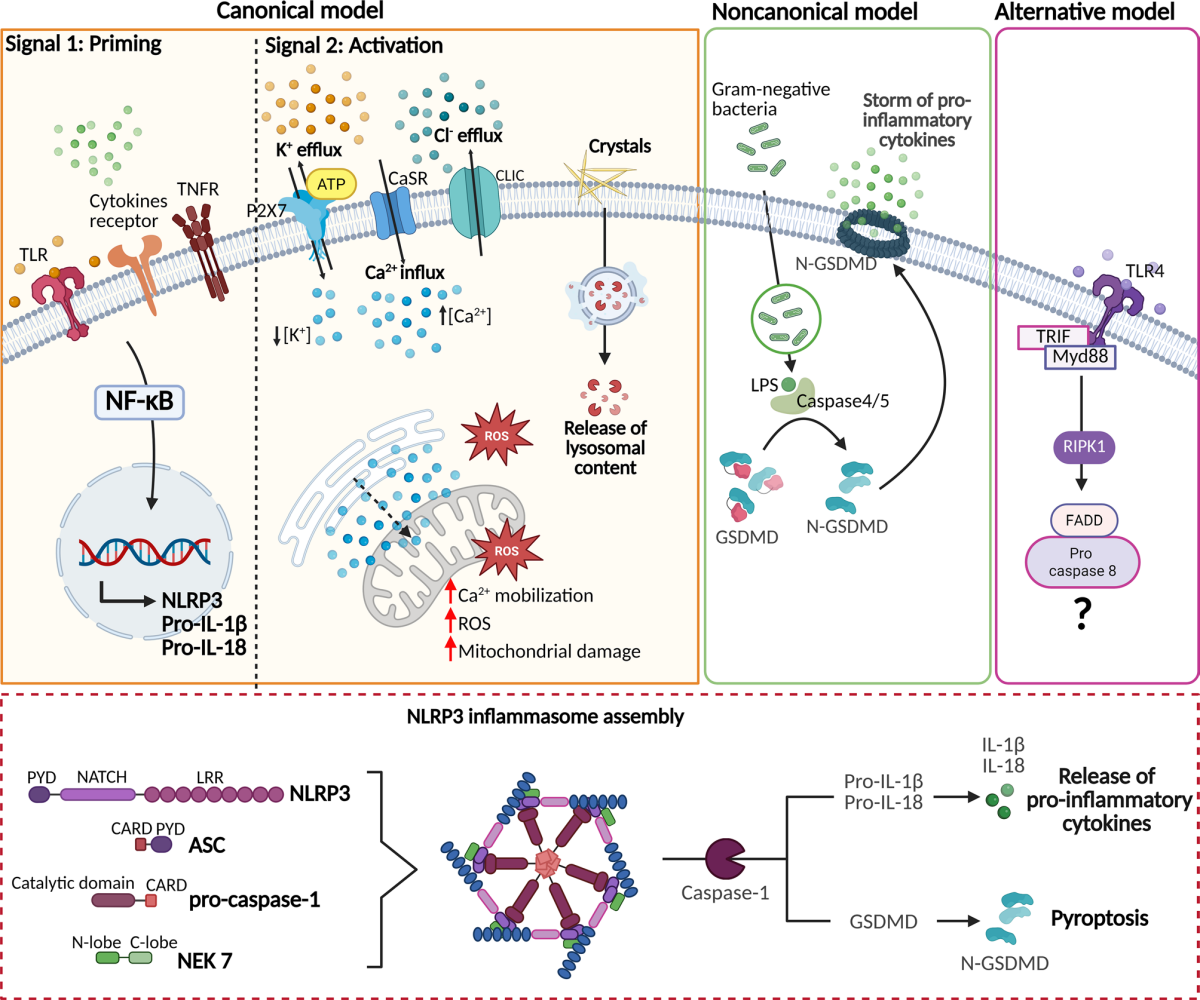
NLRP3 inflammasome activation. The NLRP3 inflammasome can be activated by canonical, non-canonical and alternative pathways. The canonical process of NLRP3 assembly requires two steps: priming and activation. Priming requires the recognition of PAMPs and DAMPs by TLRs or cytokines such as IL-1β, IL-18 or TNF which activate their signaling pathways. This step induces the direct activation of the transcription factor NF-kB at the nuclear level, leading to the transcription of the genes encoding NLRP3, pro-IL-1β and pro-IL-18. The second step involves inflammasome formation upon activation. NLRP3 is activated by a wide range of stimuli, including PAMPs or DAMPs, that in turn trigger downstream events such as ions flux (K^+^ and chloride - Cl^−^ efflux; calcium-Ca^2+^ influx), lysosomal disruption, mitochondrial dysfunction, and ROS release. Extracellular ATP binds P2X purinoceptor 7 (P2X7R), mediating K^+^ efflux. Ca^2+^ influx through the plasma membrane or its mobilization from the ER increases cytosolic Ca²⁺ levels and prompts mitochondrial alterations, contributing to NLRP3 inflammasome activation. Mitochondrial ROS and K^+^ efflux promote Cl^-^ intracellular channel protein (CLIC) membrane translocation and Cl^-^ efflux, an essential signaling event upstream of NLRP3 activation. In the noncanonical pathway, the recognition of cytosolic LPS of gram-negative bacteria by caspases-4–5 (humans) or caspase-11 (mice) promotes GSDMD cleavage and activation, triggering a storm of pro-inflammatory cytokines that can activate the canonical NLRP3 pathway. An alternative pathway of NLRP3 inflammasome is mediated by the RIPK1-FADD-CASP8 (receptor-associated protein kinase-1- Fas-associated via death domain-Caspase 8) axis. In the lower part, a schematic representation of the NLRP3 inflammasome components, their assembly and their function in danger signals recognition is shown. Once assembled, the NLRP3 inflammasome promotes the cleavage of pro-inflammatory cytokines pro-IL-1β and pro-IL-18, as well as GSDMD, into their activated forms. Created with Biorender.com

Inflammasomes are macromolecular cytoplasmic platforms that activate proinflammatory caspases upon the recognition of a variety of danger signals. Among the inflammasomes described to date, NLRP3 is the most investigated and characterized [8, 9]. The NLRP3 inflammasome is a crucial component of the innate immune system and it contains the sensor protein NLRP3, the adaptor protein apoptosis-associated speck-like protein (ASC) and the effector protein Caspase-1. The NLRP3 protein is composed of a leucine-rich repeat (LRR) domain at the C-terminus, a central NACTH domain and an N-terminal pyrin domain (PYD). Under resting conditions, the NACHT domain is masked by the LRR domain, preventing oligomerization and interaction with ASC. Following PAMPs (pathogen-associated molecular patterns) and DAMPs (damage-associated molecular patterns) sensing, conformational changes in NLRP3 allow binding between NLRP3 and ASC via homotypic PYD-PYD interactions. Through its caspase activation and recruitment domain (CARD), ASC subsequently promotes the recruitment of pro-caspase 1 monomers, resulting in the formation of active caspase-1 through proteolytic self-cleavage. In turn, Caspase-1 cleaves the cytokine precursors and the C-terminus of Gasdermin D (GSDMD), leading to the release of interleukin-1β (IL-1β) and interleukin-18 (IL-18), as well as N-Gasdermin (N-GSDMD), which are responsible for inflammatory signaling and pyroptotic cell death, respectively [10]. The NLRP3 inflammasome can be activated by canonical, noncanonical and alternative pathways (Fig. 1), making it difficult to define a universal mechanism for its assembly [11]. Canonical activation of the NLRP3 inflammasome follows a two-step model including a ‘priming’ phase followed by an ‘activation’ phase. Priming requires the recognition of PAMPs and DAMPs by Toll-like receptors (TLRs) or cytokines such as IL-1β, IL-18 and tumor necrosis factor (TNF) which activate their signaling pathways. This step induces the direct activation of the transcription factor kappa-light-chain-enhancer of activated B cells (NF-κB) at the nuclear level, leading to the transcription of the genes encoding NLRP3, pro-IL-1β and pro-IL-18, and an increase in their expression [12–14]. Priming also involves the induction of post-translational modifications (PTMs) such as ubiquitylation, SUMOylation, and phosphorylation [15]. Additionally, interfering RNAs can regulate the transcriptional expression of NLRP3 [16]. The second step involves inflammasome formation upon activation. NLRP3 is activated by a wide range of stimuli. However, the mechanisms by which NLRP3 detects cellular stress and how these pathways induce inflammasome formation and activation remain poorly understood. Upstream signals that activate NLRP3 are often interconnected and overlapping, and include ion flux, lysosomal disruption, extracellular adenosine triphosphate (ATP), crystallization, mitochondrial dysfunction and ROS production, metabolic disturbances, and changes in sphingolipid metabolism [17].

In the noncanonical pathway, the NLRP3 inflammasome can be triggered independently of caspase-1. In detail, caspase-11, in mice, and its human orthologues, caspases 4–5, can recognize cytosolic lipopolysaccharide (LPS) of gram-negative bacteria inducing the NLRP3 inflammasome assembly. Caspase-11 promotes pyroptosis by cleaving GSDMD, which forms pores and triggers a storm of pro-inflammatory cytokines that can activate canonical NLRP3 pathway [18]. In humans, caspase-4 is commonly considered the functional homolog of caspase-11, as it is involved in both the release of IL-1β and pyroptosis after LPS stimulation [19]. Caspase-11 is generally poorly expressed in mice, so a priming signal is required to increase its expression [20]. In contrast, a priming signal is not necessary to activate the noncanonical pathway in humans because caspase-4 is highly expressed [21]. An alternative pathway of NLRP3 inflammasome activation does not require the second signal expected from the “two-step” paradigm. This model is mediated by the RIPK1-FADD-CASP8 axis and takes place independently of K^+^ efflux and P2X7R activation [22].

NLRP3 activation is tightly regulated by several accessory proteins, one of which is the never in mitosis A (NIMA)-related kinase 7 (NEK7). NEK7 drives NLRP3 oligomerization and the maturation of proinflammatory cytokines downstream of K^+^ efflux. He et al. reported that NEK7 deficiency impairs NLRP3 inflammasome assembly and ASC speck formation [23], whereas cryo-EM studies revealed that NEK7 binds adjacent NLRP3 subunits, facilitating inflammasome oligomerization [24]. However, whether NEK7 is strictly essential for NLRP3 activation remains under debate. Other regulators include thioredoxin-interacting protein (TXNIP) and mitochondrial antiviral signaling (MAVS) which bind to NLRP3 in response to redox alterations and RNA viruses, respectively [25, 26]. The stress granule protein DEAD-box helicase 3 X-linked (DDX3X) has been shown to interact with NLRP3 and promote the pyroptotic ASC specks [27]. Similarly, MARK4 (microtubule affinity-regulating kinase 4) physically binds NLRP3 and drives it to the microtubule organizing center, facilitating the inflammasome assembly [28]. A recent report identified promyelocytic leukemia protein (PML) as a negative regulator of NLRP3. Specifically, PML forms a trimeric complex with NLRP3 and P2X7R at ER-mitochondria contact sites, thereby restraining inflammasome activation and the release of pro-inflammatory cytokines within the tumor microenvironment [29]. Further studies are warranted to clarify the molecular mechanism governing NLRP3 interactions and how these interactions are regulated in their respective context.

## Impact of radiation on NLRP3 inflammasome activation

**Fig. 2:**
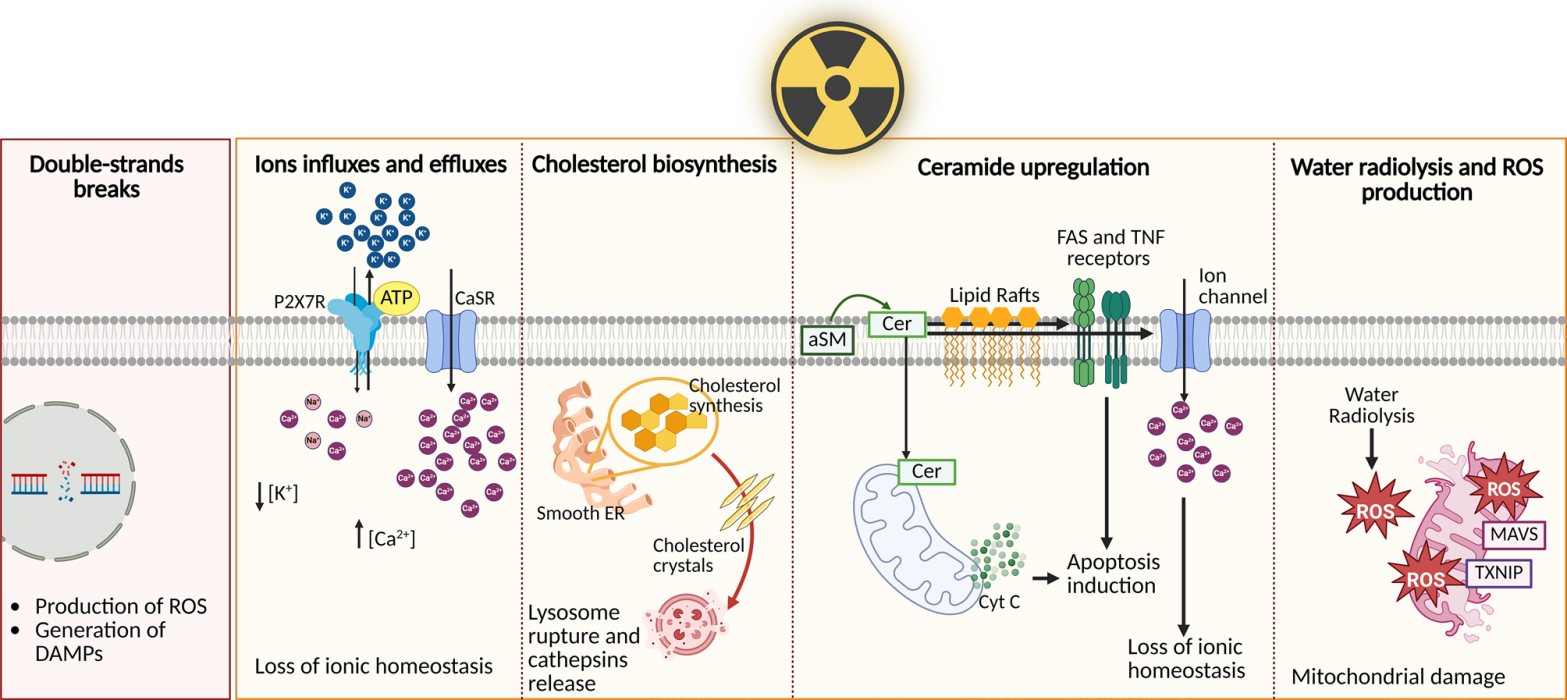
Mechanisms by which radiation triggers NLRP3 inflammasome activation. Radiation can produce trigger signals for NLRP3 inflammasome assembly: DSBs which stimulate NLRP3 activation indirectly through ions, DAMPs and ROS modulation; ions fluxes, such as potassium efflux and calcium influx; cholesterol biosynthesis and anincrease in cholesterol crystals, which can lead to lysosome breakdown; upregulation of ceramide, which can stimulate Fas and TNF receptors, translocate to the mitochondrion and determine the release of cytochrome C, as well as modulate certain ion channels; and water radiolysis and ROS production, which are responsible for mitochondrial dysfunction leading to the release of further DAMPs. These signals prompt NLRP3 inflammasome activation and assembly, resulting in the release of pro-inflammatory cytokines and the induction of pyroptosis. Created with Biorender.com

Radiation can activate the NLRP3 inflammasome through several mechanisms (Fig. 2). Radiation causes DNA damage, which activates repair mechanisms. Following DSBs, the ataxia telangiectasia mutated (ATM) protein initiates a series of responses to repair the damage. In this process, ATM promotes the activation of NF-κB in the cytoplasm [2], potentially inducing NLRP3 transcription. Moreover, DNA damages produce ROS and DAMPS [30] that could mediate the indirect activation of NLRP3.

K^+^ efflux and Ca^2+^ flux related to radiation have been extensively studied. Ca^2+^ influx into the cytosol can occur through the opening of plasma membrane channels or via release from the intracellular Ca^2+^ pool in the ER. K^+^ efflux serves as a counter regulator of Ca^2+^ influx. As a result, Ca^2+^ influx and K^+^ efflux are often found to be coordinated in the activation of NLRP3 [31]. ATP is another key signaling molecule involved in radiation-induced biological effects [32]. The activation of the P2X7R by ATP triggers a weak influx of Ca^2+^ and sodium (Na^+^), along with concurrent K^+^ efflux [33]. Low intracellular potassium levels have been shown to activate the NLRP3 inflammasome in THP-1 cells and bone marrow-derived macrophages in vitro [34].

Radiation can also directly induce cholesterol biosynthesis [3]. Phagocytosis of cholesterol crystals leads to lysosomal rupture and the release of particles into the cytoplasm. Moreover, radiation has been shown to directly cause lysosomal destabilization [35]. Both cholesterol crystals and radiation are known to induce lysosomal rupture, triggering NLRP3 activation [36]. Furthermore, cathepsins released from ruptured lysosomes play a critical role in NLRP3 activation following radiation exposure [37]. However, individual knockouts of cathepsins B, X, L, or S had little impact on NLRP3 activation, suggesting that cathepsins may function redundantly in the activation of NLRP3 signaling [38].

Recent studies have also indicated that radiation-induced upregulation of cellular ceramide [39] serves as a second messenger in the initiation of intrinsic apoptosis. Ceramide plays multiple pathophysiological roles in radiation induced NLRP3 activation. Ceramide-mediated activation of cathepsin D has been shown to link TNF α-induced acid sphingomyelinase to Bid-related mitochondrial apoptosis [40]. Another research has identified plasma membrane ion channels as novel targets of ceramide, including those responsible for Ca^2+^ influx and K^+^ efflux [41]. These findings demonstrate that many mechanisms of NLRP3 activation involve either Ca^2+^ or K^+^ flux.

Most radiation-induced damage results from water radiolysis, as more that 80% of the cell mass or tissue is composed of water. Ionizing radiation causes water radiolysis, leading to the production of ROS, which are the primary contributors to radiation-induced tissue damage. The NLRP3 protein contains a highly conserved disulfide bond that links the PYD domain to the nucleotide-binding site domain, making it highly sensitive to changes in the redox state [42]. Emerging evidence emphasize the role of mitochondria and mitochondrial oxidative stress in mediating radiation-induced injury [43, 44]. Radiation-induced mitochondrial dysfunction, along with the release of mitochondrial ROS (mtROS) and DNA into the cytoplasm, are key upstream regulators of NLRP3 activation [45]. PINK1/Parkin-mediated mitophagy is essential for removing damaged mitochondria, thereby attenuating both inflammation and oxidative stress [46, 47]. Maintaining a balance in mitochondrial fusion and fission is critical in mitochondrial health and homeostasis [48]; imbalances in these mechanisms elevate ROS levels, which perpetuate mitochondrial dysfunction through a self-amplifying feedback loop and, potentially, NLRP3 pathways activation [44, 49]. mtROS production and mitochondrial DNA (mtDNA) damage, as a consequence of radiation, reduce the transcription of mtDNA-encoded electron transport chain (ETC) complexes, impairing ETC activity and promoting further mtROS production [50]. As previously mentioned, radiation can directly act on DNA, causing strand breaks; however, it can also trigger the activation of mitochondrial cell death signaling pathways. The release of cytochrome C, the activation of Ca^2+^/calmodulin-dependent protein kinase II (CaMKII) axis and intracellular iron overload, which are involved in apoptosis, necroptosis, and ferroptosis respectively, represent mechanisms that contribute to a self-perpetuating cycle of oxidative stress and inflammatory cascade [51–53].

Ionizing radiation can also produce ozone, which has been associated with cardiovascular injury [54, 55]. Ozone-induced oxidative stress triggers inflammasome activation, leading to the release of IL-1 and other inflammatory cytokines [56]. ROS may act both as “kindling” and as the “bonfire” for NLRP3 inflammasome activation. ROS function as redox signaling messengers that initiate NLRP3 activation [57]. Once the NLRP3 inflammasome is activated, ROS and inflammatory cytokines are generated, creating a feedback loop that amplifies the response. Excessive ROS lead to intracellular and extracellular oxidative stress, causing damage to nucleotides, lipids, and proteins. Although there is substantial evidence supporting the role of radiation-induced ROS in activating NLRP3 inflammasomes, the precise mechanisms remain largely unclear. Two proteins, TXNIP and MAVS, have been identified as potential mediators of ROS-induced NLRP3 activation [26, 58]. Additionally, radiation-induced damage can provoke mitosis and pyroptosis, resulting in the release of DAMPs and further activation of the NLRP3 inflammasome. Studies have shown persistent expression of NF-κB in patient neck vessels even 10 years after radiation exposure, which promotes NLRP3 transcription [59]. These findings suggest a potential link between NF-κB-dependent NLRP3 activation and chronic fibrosis.

## NLRP3 inflammasome in radiation-induced cardiovascular diseases

**Fig. 3:**
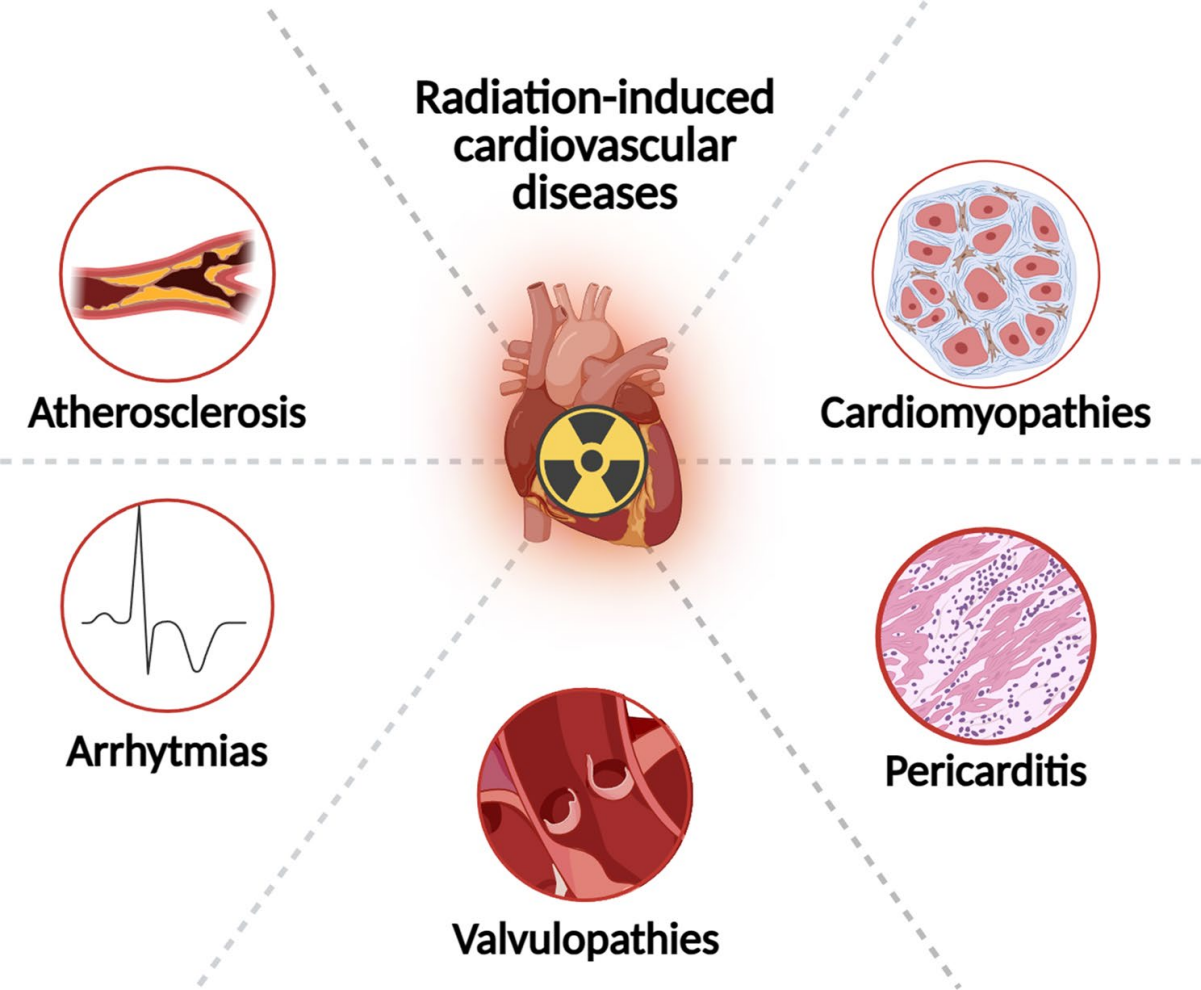
NLRP3 inflammasome involvement in radiation-induced cardiovascular diseases. Created with Biorender.com

RICVD represents a growing problem in a population with an increasing number of cancer survivors. When the irradiation area includes the heart, injury can occur at the level of the pericardium, myocardium, vascular system, valves, resulting in a heterogeneous and difficult to diagnose syndrome (Fig. 3). Clinical and preclinical research has made great strides in this area, leading to the development of radiotherapy protocols that involve less healthy tissues and lower radiation doses. However, many cancer patients undergo radiation therapy, which increases their risk of cardiovascular disease.

In the last ten years, the overexpression of NLRP3 and the activation of the inflammasome have been associated with radiation-induced lung inflammation, oral mucositis and skin lesions [60], as well as cardiovascular diseases [7]. A study by Halle et al. shed the light on the role of the transcription factor NF-κB in cardiovascular complications of radiations. Researchers found dysregulation of the NF-κB signaling pathway in irradiated human arteries compared to those that were nonirradiated from the same patient [59]. NF-κB is known to be involved in the inflammatory response and in the regulation of various genes implicated in inflammation, including the NLRP3 gene. The scientific community has recently focused on mitochondrial dysfunction and oxidative stress as key contributors to radiation-induced cardiac injuries. As mitochondria are the main energy source for cardiomyocytes, it is not surprising that mitochondrial damage, impaired metabolism and mitophagy, and release of mtDNA contribute to inflammation and progression of RICVD [43, 61–63].

Currently, it is well-accepted that the mechanism of RICVD rise, and progression require various factor that interact with each other, including endothelial cell injury, ROS production, apoptosis and microRNAs. The early damages of RT are mainly related to the activation of chronic and prolonged inflammation, whereas the later effects are associated with inflammation and fibrosis. RICVDs are a dynamic a progressive class of pathologies, in which the NLRP3 inflammasome may have some contribute. Among the complications in patients treated with radiotherapy, accelerated atherosclerosis and cardiac fibrosis, which contributes to various cardiac diseases, represent the most relevant. Both disorders are characterized by chronic inflammation, and the NLRP3 inflammasome could be the key factor for their initiation and worsening. The production of pro-inflammatory cytokines, in fact, fuels inflammation, sustaining its chronicity, and promoting fibrotic processes.

### NLRP3 in radiation-induced accelerated atherosclerosis

Radiation-induced coronary artery diseases are the leading cause of RICVD, accounting for approximately 85% of cases [64]. Radiation exposure damages the endothelium of vessels and triggers a long-lasting inflammatory response. This prolonged inflammation results in a series of complications that lead to the onset of atherosclerosis [65]. Radiation-induced coronary artery disease affects younger patients, and it is closely related to both the area and the dose of irradiation. The strongest evidence for this category of disease has been collected through studies of breast cancer and Hodgkin lymphoma survivors, due to their high 5-year survival rate [66]. Currently, the involvement of NLRP3 in radiation-induced coronary artery disease has not yet been demonstrated, and the literature on this topic is quite limited. However, as an inflammatory disease, the role of NLRP3 in the pathogenesis of atherosclerosis has been widely investigated.

Numerous studies have reported that IL-1β is involved in both the onset and the progression of atherosclerosis; however, randomized clinical trials have confirmed its therapeutic relevance only in the last decade [67, 68]. A double-blind phase-II study supported the use of canakinumab, a monoclonal antibody targeting IL-1β, in the management of inflammation-driven atherosclerosis. In a cohort of men and women with well-controlled diabetes mellitus and high cardiovascular risk, canakinumab significantly reduced both IL-6 and C-reactive protein levels, confirming the therapeutic relevance of targeting inflammation [68]. Building on these results, the CANTOS trial evaluated the potential of canakinumab in more than 10,000 subjects with a history of myocardial infarction and reported a lower rate of recurrent cardiovascular events in the treated group than in the placebo group [67]. Moreover, in humans, increased levels of IL-1β are detected in atherosclerotic arteries compared to healthy tissues and these levels were correlated with the severity of the condition [69]. Coherently, NLRP3 was strongly expressed in the aorta of patients with coronary atherosclerosis and its expression levels correlated with the severity of the disease [70]. In a related study, Paramel Varghese et al. evaluated the mRNA levels of NLRP3 and NLRP3 inflammasome components in the atherosclerotic plaques of myocardial infarction patients compared to nonatherosclerotic vessels. They found higher levels of NLRP3, ASC, Caspase-1, IL-1β and IL-18 mRNA in the pathological samples compared to healthy arterial samples and, interestingly, this expression was more pronounced in patients with severe symptoms [71].

The role of NLRP3 in radiation-induced coronary artery diseases has not yet been elucidated, but several studies have highlighted how inflammatory mechanisms are key actors in the onset and progression of these pathological conditions. An experimental study on hypercholesterolemic rabbit model demonstrated that single-dose irradiation of the aorta accelerated the formation of atherosclerotic plaques [72]. Similarly, Steward and colleagues established a murine model to study radiation-induced atherosclerosis. Irradiation of atherosclerosis-prone apolipoprotein E knockout (ApoE^−/−^) mice with high single dose or fractionated schedules affected plaque quality, in terms of macrophage infiltration, collagen content and intraplaque hemorrhage. Although no changes in plaque burden were observed, plaques shifted toward an inflammatory phenotype characterized by the presence of granulocytes, potentially increasing vulnerability to plaque rupture [73, 74]. About a decade later, the same group investigated the impact of radiation on pre-existing atherosclerotic lesions, finding smaller plaques, but particularly rich in macrophages polarized toward the pro-inflammatory state in irradiated ApoE^−/−^ mice [75]. Furthermore, the role of radiation in promoting the accumulation of macrophage-rich lesions has been demonstrated in studies with low density lipoprotein receptor knockout (LDLR^−/−^) mice irradiated with 10 Gy and followed by bone marrow reconstitution [76]. Altogether, these findings suggest that radiation could lead to a chronic inflammatory cascade, contributing to the development and the progression of atherosclerosis with increased inflammation and intraplaque hemorrhage.

Multiple atherogenic factors induced by radiation have been shown to activate the NLRP3 inflammasome, leading to endothelial damage, vascular smooth muscle cells (VSMCs) proliferation and subsequent atherogenesis. Single and fractionated X-ray irradiation of the coronary artery alters endothelial connexin expression and increases gap junctional communication and hemichannel functions [77]. Connexin hemichannels are generally closed and their unregulated opening leads to ion flux alterations, ATP release and loss of cell homeostasis, all of which are danger signals that can trigger the activation of the NLRP3 inflammasome [77]. Moreover, radiation directly affects the vasculature by inducing endothelial cell death and senescence. Specifically, most endothelial cells undergo mitotic cell death in response to fractionated radiotherapy protocols, while higher doses trigger apoptotic cell death [78]. Cell death by mitotic catastrophe, as well as apoptosis, leads to the release of DNA damage signals, ATP and other DAMPs which can contribute to the activation of the inflammasome and, therefore, stimulate an inflammatory response [79]. Radiation disrupts the inflammatory balance in the vascular microenvironment by inducing the activation and expression of selectins and integrins, which are responsible for the recruitment of immune cells from the circulation to the inflamed area [80]. Radiation-induced activation of endothelial cells could thus be responsible for modulating VSMCs proliferation, contributing to the progression of atherosclerosis [81]. Studies on this point are rather conflicting, probably due to very different irradiation protocols [82]. As innate immune cells, macrophages are recruited to the irradiated area and play an important role in the development of radiation injury. Notably, radiotherapy can strongly affect macrophage polarization towards the M1 phenotype, shaping the pro-inflammatory response [83]. Despite the well-established role of inflammation, particularly chronic inflammation, few studies to date have evaluated the role of the NLRP3 inflammasome and the molecular mechanisms underlying radiation-induced coronary artery diseases, highlighting a significant gap in the scientific literature.

### NLRP3 in radiation-induced cardiomyopathies

Cardiomyopathy represents a group of diseases affecting the heart muscle. Cardiac fibrosis is one of the key pathological hallmarks of cardiomyopathy [84], and radiation exposure can promote its progression and contribute to the earlier onset of cardiomyopathy [85, 86]. The main effectors of radiation-induced fibrosis are macrophages which maintain a chronic inflammatory state in damaged tissue [87]. The initiation stage is driven by M1-polarized macrophages, which are responsible for the release of transforming growth factor-beta (TGF-β), chemokines and other mediators which, in response to injury, promote fibroblast activation, proliferation and differentiation into mature myofibroblasts. These fibrogenic effector cells produce large amounts of extracellular matrix proteins, such as collagen. Polarized M2 macrophages, in a subsequent phase and in an attempt to heal the damaged tissues, drive the progression of fibrosis by secreting cytokines and promoting the further transformation of fibroblasts into myofibroblasts [88]. Radiation-induced cardiac fibrosis is a potentially lethal complication with an incidence of up to 80%, depending on factors such as the radiation dose and patient comorbidities [85]. This form of fibrosis progresses gradually over the years, often manifesting clinical symptoms long after radiotherapy [86]. Myocardial fibrosis is mediated by cardiac fibroblasts which derived primarily from mesenchymal precursors and resident cardiac fibroblasts, with potential contributions from bone marrow stem cells or other tissues [89].

The role of NLRP3 in different types of fibrosis is partially recognized. In the context of cardiac fibrosis, researchers reported that NLRP3 was expressed in cardiac fibroblasts and that its expression increased upon TGF stimulation [90, 91]. Interestingly, only the NACHT domain of NLRP3 was required for R-smad activation in response to TGF-β, promoting the differentiation of myofibroblasts independently of inflammasome assembly [90, 91]. On the other hand, the persistent localization of NLRP3 in the mitochondria of cardiac fibroblasts and the crucial role of ROS in TGF-β signaling, supported the hypothesis that NLRP3 modulates mtROS levels to influence smad activation and fibrotic gene expression [90]. From a critical perspective and considering that the expression of NLRP3 is significantly lower in fibroblasts than in immune cells, non-canonical inflammasome activation mechanisms should be investigated. Another study revealed that MCC950, a well-known specific NLRP3 inhibitor, rescued myocardial damage and reduced cardiac fibrosis in a murine model of myocardial infarction [92].

One of the few studies investigating the role of the NLRP3 inflammasome in radiation-induced heart disease is that of Mezzaroma and colleagues. They investigated the involvement of IL-1 in XRT-induced cardiomyopathy, which is characterized by myocardial and pericardial fibrosis. When irradiated, mouse models lacking IL1R1(interleukin 1 receptor, type 1) were resistant to radiation damage, while wild type models developed alterations in myocardial and systolic function. Coherently, but less effectively than genetic inhibition, treatment with Anakinra limited myocardial injury [93]. Despite it has not been studied at the cardiac level, the NLRP3 inhibitor NXC736 has shown benefit sin the inhibition of collagen deposition and inflammatory infiltration in radiation-induced lung fibrosis [94].

### NLRP3 in radiation-induced pericarditis

Radiation-induced acute pericarditis is a short-term complication of high-dose radiation, but, owing to innovative treatment techniques, it is considered a very rare side effect. RT can cause inflammation of the pericardium and activation of the immune system, which, when chronic, leads to the replacement of the pericardial adipose tissue with collagen and fibrin. However, no clear evidence for the role of NLRP3 in radiation-induced pericarditis has been reported, while an involvement of NLRP3 in the pathogenesis of pericarditis has been assumed in recent years. According to the ESC guidelines for the management of pericarditis, colchicine is recommended as first line therapy in combination with non-steroidal anti-inflammatory drugs - NSAIDs [95]. Notably, colchicine inhibits microtubule assembly and the presentation of danger signals to NLRP3, impairing inflammasome activation [96]. Another proof, although indirect, derived from the AIRTRIP Randomized Clinical Trial, a double-blind and placebo-controlled trial including 21 patients with recurrent pericarditis. The risk of pericarditis recurrence was reduced in the anakinra-assigned group compared to placebo group [97]. Confirming its involvement in pericarditis, Mauro’s group reported the presence of NLRP3, ASC and caspase-1 expression in pericardial biopsies from pericarditis patients. Moreover, they demonstrated increased expression of IL-1α and IL-1β in the pericardium of mouse models of pericarditis compared with controls. The inhibition of IL-1 in mice with pericarditis turned off the NLRP3-mediated inflammation, ameliorating pericardial effusion and thickening [97]. It is possible to assume that NLRP3 could play a central role also in radiation-induced pericarditis.

### NLRP3 in radiation-induced valvular heart diseases

Radiation-induced heart valve damage is characterized by valve fibrosis and calcification and evolves from asymptomatic to clinically significant disease in a range of 10–20 years after radiation exposure [98]. The pathogenesis of radiation-induced valvular heart diseases is not clearly understood; however, radiation therapy is known to activate TGF-β and collagen deposition [90, 91]. It has been reported that irradiation induces an osteogenic phenotype in isolated human aortic valve interstitial cells, potentially involving inflammatory pathways [99, 100]. In particular, the cytokines released by immune cells infiltrating the valve drive the differentiation of interstitial cells into myofibroblasts and osteoblastic-like cells in valve stenosis. The inhibition of NLRP3 by CY-09 in vivo downregulates the release of pro-inflammatory cytokines and osteogenic markers, resulting in improved aortic valve function and a decrease in calcification deposits [99].

### NLRP3 in radiation-induced arrhythmias

Cardiac arrhythmias are recognized as a significant complication of thoracic radiotherapy. A recent study enrolling 360 patients undergoing thoracic radiotherapy, primarily for lung cancer, reported a significant increase in the prevalence of supraventricular arrhythmias after RT [101]). Despite emerging evidence, significant gaps in our knowledge mean that the precise mechanisms underlying radiation-induced arrhythmias remains limited. Direct radiation injury and fibrosis of conduction system represent a possible mechanism of the onset of arrhythmia, both tachyarrhythmia and bradyarrhythmia. However, NLRP3 inflammasome activation has emerged as a key contributor to pathological remodeling processes of cardiac arrhythmias. According to the study of Li and colleagues, NLRP3 inflammasome activity was enhanced in the cardiomyocytes of patients with atrial fibrillation (most common cardiac arrhythmia) [102]. In murine cardiomyocytes, NLRP3 inflammasome activation predisposes to ectopic electrical activity and drives both structural and electrophysiological remodeling (e.g. fibrosis and altered potential duration) that support the initiation and the progression of atrial fibrillation [102]. In this model, NLRP3 triggering was correlated to disrupted intracellular calcium homeostasis resulting from ryanodine receptor 2 (RyR2) upregulation. This led to abnormal release of calcium and occurrence of ectopic electrical impulses, which sustain arrhythmogenesis [102]. NLRP3 activation in cardiomyocytes also enhances a specific potassium current (I_Kur_), shortening the atrial refractory period and facilitating the formation of re-entry substrates-another mechanism that maintains atrial fibrillation. In parallel, the excessive NLRP3 activity may increase caspase-1 cleavage and pyroptosis, leading to the release of additional danger signals. It is therefore clear that NLRP3 mediates several signaling pathways that act in concert to promote arrhythmogenesis [102]. Aligned with the work already discussed, the pro-inflammatory cytokine IL-1β has been linked to atrial fibrillation rise and arrhythmogenesis. Acute exposure of IL-1β to a murine cardiomyocyte cell line activates NLRP3-dependent pathways and upregulates CaMKII/RyR2 axis, sensitizing cardiomyocytes to spontaneous Ca^2+^ fluxes and electrophysiological alterations [103] These findings highlight the central role of NLRP3 in arrhythmogenesis, yet its contribution to radiation-induced arrhythmias remains unexplored.

## Modulating the NLRP3 inflammasome as a therapeutic option for radiation-induced cardiovascular diseases

Given the known involvement of NLRP3 in various pathologies, the development of specific inhibitors is urgently needed. To our knowledge, no specific inhibitors of NLRP3 have been tested for RICVD. Therefore, this section includes findings related to other organ injuries or, more generally, to cardiovascular diseases, to highlight the potential efficacy of these approaches in ameliorating radiation-induced cardiovascular disease.

The use of inflammatory mediators’ inhibitors has shown promising results. A study reported beneficial effects of the IL-1 receptor antagonist anakinra in ApoE^−/−^ irradiated mice. Specifically, the administration of the inhibitor decreased the expression of pro-inflammatory mediators in the artery of mice [104]. Despite its several limitations, this study offers interesting insights, especially considering that anakinra is already being used in clinical trials focused on pericarditis, heart failure and myocardial damage [105–107]. As previously mentioned, the CANTOS trial reported a lower rate of recurrent cardiovascular events and cardiovascular mortality in the canakinumab-treated group than in the placebo group [67].

Colchicine is an anti-inflammatory drug mainly used to treat gout and cardiovascular inflammation. It works by inhibiting microtubule polymerization, which interferes with the interaction between NLRP3 and ASC, thereby preventing inflammasome assembly. Colchicine is recognized as a first-line treatment for pericarditis. Given its favorable safety profile and proven efficacy, it is not surprising that it is also being evaluated as a potential treatment for other cardiovascular diseases. A number of clinical trials (LoDoCo, LoDoCo2, COLCOT) have validated the efficacy of colchicine in reducing cardiovascular events in patients with coronary artery disease [108, 109] and myocardial infarction [110]. To date, there are no current recommendations for the use of colchicine in the prevention of RICVD. However, growing evidence, although indirect, supports a preventive effect of colchicine in the development of long-term side effects of radiotherapy. Resveratrol is a natural nonflavonoid polyphenol which acts on the NF-κB gene via SIRT1 activation, leading to the transcriptional repression of inflammation-related genes. The group of Liu demonstrated that resveratrol reduced the expression of IL-1β in the thymus and spleen of irradiated mice [111]. NLRP3 transcription is NF-κB-dependent, suggesting that IL-1β alteration by resveratrol occurs via NLRP3 pathway modulation [111].

The design and development of small molecules that specifically inhibit the NLRP3 inflammasome could represent a promising strategy for the treatment and prevention of RICVD. Among the inhibitors reported in the literature, MCC950 was identified in 2003 [112] and has shown remarkable potential in pre-clinical studies. MCC950 is a well-established NLRP3 inhibitor which binds to the Walker B motif of the NACTH domain of NLRP3 blocking the ATP to ADP (adenosine diphosphate) hydrolysis [113, 114]. Both in vitro and in vivo studies have demonstrated its benefits in improving cardiac function in various cardiovascular diseases, including atherosclerosis, myocardial infarction, and heart failure [115]. Despite its high efficacy and NLRP3 specificity in preclinical studies, its pharmacokinetic profile makes it unsuitable for clinical use. In fact, during a phase II clinical trial for rheumatoid arthritis, MCC950 was found to induce liver toxicity, the cause of which is still unknown [116]. The development of MCC950 and its promising effects paved the way for the design of NLRP3 analogues and inhibitors [117], further establishing NLRP3 as a key pharmacological target within the scientific community. One example of MCC950 analogue is IFM-2427, which was evaluated in a phase I healthy volunteers trial exploring its potential in gout, Crohn’s disease, and coronary artery disease [118]. INF4E is a potent NLRP3 inflammasome inhibitor; specifically, it inhibits caspase-1 and NLRP3 ATPase activities [118, 119]. Mastrocola et al. demonstrated the efficacy of INF4E in myocardial ischemia-reperfusion injury, showing that it significantly reduced infarct size and preserved cardiac function in rats [119, 120]. 16673–34 − 0 is a glyburide derivative lacking a cyclohexylurea moiety that inhibits NLRP3 inflammasome assembly. Marchetti et colleagues demonstrated that 16673–34 − 0 reduced caspase-1 activity and cell death in cardiomyocytes and limited the infarct size after myocardial ischemia–reperfusion in vivo [121]. Similarly, the same group reported that 16673–34 − 0 improved cardiac function in a model of coronary artery ligation [121]. Moreover, the NLRP3 inhibitor showed significant ability to reduce pericardial thickening and pericardial effusion in mice with acute pericarditis [122]. 16673–34 − 0 is a compound that can improve cardiac function and reduce inflammation in cardiovascular disease, potentially also that caused by radiations. Analogs of this compound have been developed and found to be more effective in inhibiting NLRP3 [123]. Another NLRP3 inhibitor reported in the literature to be beneficial in cardiovascular diseases is Bay 11-7082. It is a synthetic kappa B kinase b inhibitor, and it alkylates the cysteine residue in the ATPase domain of NLRP3, blocking the inflammasome formation. Treatment with Bay 11-7082 before reperfusion in a mouse model of ischemia-reperfusion attenuated immune cell infiltration and cardiac injury [124]. Similar results were reported independently by Kim’s group and Qiu’s group, confirming the efficacy of the inhibitor in reducing inflammation and cardiomyocyte cell death in rat models [125, 126]. The results showed clear cardioprotective effects but given the compound’s ability to act on different pathways, the precise mechanism of action is unclear. As Bay 11-7082, also OLT117 inhibits NLRP3 by blocking its ATPase domain [127, 128]. Its potential in treating inflammatory disease has been tested in several models of disease, both in vivo and in vitro. One of these studies revealed that OLT1177 reduced infarct size and preserved cardiac systolic function in a mouse model of ischemia reperfusion injury [129]. Of note, this compound showed a safe profile also in clinical trials [130, 131].

Other inhibitors, such as LGM2605 [132] and CB001 [133], have been demonstrated to have radioprotective effects and to reduce tissue damage induced by radiations. Their direct effect on NLRP3 inflammasome has not yet been adequately reported, but it has been showed that LGM2605 reduces NLRP3 expression in lung tissues after radiation exposure [132] and that CB001 exerts its radioprotective effect via the modulation of NLRP3 signaling [133].

Taken together, these results highlight the potential efficacy of inhibitors of NLRP3 or its downstream pathways in improving or preventing radiation-induced cardiovascular disease.

## Concluding remarks

The NLRP3 inflammasome has emerged as the most studied and relevant platform in the context of radiation-induced cardiovascular toxicity. However, other inflammasome complexes—such as absent in melanoma 2 (AIM2), NLR family CARD domain containing 4 (NLRC4), NLR family pyrin domain containing 1 (NLRP1), and pyrin—have also been implicated in inflammatory and stress-related responses, especially in infectious or autoimmune conditions. While their roles in radiation-induced damage remain poorly characterized, understanding their distinct activation mechanisms and pathological associations could broaden our perspective on inflammasome-driven cardiovascular injury. As summarized in Table 1, NLRP3 stands out for its ability to sense multiple danger signals relevant to ionizing radiation—including ROS, mitochondrial dysfunction, DNA damage, ion fluxes, and DAMPs release—making it a particularly compelling target in sterile inflammation and cardiac injury. This comparative view further reinforces the rationale for selectively targeting NLRP3 in radiation-induced cardiovascular diseases.

NLRP3 is therefore a key mediator of radiation-induced cardiotoxicity, contributing to the worsening of long-term outcomes and quality of life in cancer survivors. With continuous advances and innovative approaches in the field of oncology, targeting the inflammasome as a preventive strategy is becoming increasingly feasible. On the other hand, the potential consequences of NLRP3 inhibition including interference with homeostatic functions, such as tissue repair and pathogen clearance, must also be carefully evaluated [134–136].

Radiotherapy is an effective and widely used antitumor treatment in clinical practice; however, its side effects cannot be overlooked. Currently, the approach to preventing side effects of radiotherapy involves the use of fractionated-dose protocols, whereby patients are exposed to lower doses of radiation over several consecutive days. Prevention is the ideal strategy, which is why over the years several radiation protectors have been developed and tested [137, 138]. Unfortunately, according to the guidelines, only a few of them are recommended for different circumstances [139]. This further highlights the urgency of developing new effective treatments for radiotherapy-induced cardiotoxicity. A comprehensive analysis of the NLRP3 inflammasome and its role in radiation-induced cardiovascular diseases could provide valuable insights and establish the basis for innovative therapeutic approaches.

**Table 1:** Relevance and potential involvement of inflammasome complexes (NLRP3, AIM2, NLRC4, NLRP1 and Pyrin) in radiation-dependent damage and cardiovascular pathology

Inflammasome	Sensor	Activator stimuli	Effector caspase	Relevance to RT or CV pathology
NLRP3	NLRP3	ROS, K⁺ efflux, ATP, DAMPs, lysosomal rupture	Caspase-1	Strongly implicated in cardiac fibrosis, atherosclerosis, and RT-induced inflammation [7, 60, 140].
AIM2	AIM2	Cytosolic dsDNA	Caspase-1	Activated by DNA damage; possible role in radiation-induced inflammation, but no direct link to cardiovascular injury yet [141].
NLRC4	NLRC4	Bacterial flagellin, T3SS components	Caspase-1	Involved in sepsis and infection-triggered myocarditis; not linked to sterile RT damage [142].
NLRP1	NLRP1	Bacterial toxins, dsRNA	Caspase-1	Some evidence in cardiomyocyte stress and aging, but poorly explored in radiation context [143, 144].
Pyrin	Pyrin	Rho GTPase disruption	Caspase-1	Associated with autoinflammatory syndromes; potential link to vascular inflammation via IL-1β release, not directly studied in RT [145].

## Data Availability

Not applicable.

## Abbreviations

ADP: Adenosine diphosphate
AIM2: Absent in melanoma 2
ApoE^−/−^: Apolipoprotein E knockout
ASC: Apoptosis-associated speck-like protein
ATM: Ataxia telangiectasia mutated protein
ATP: Adenosine triphosphate
Ca^2+^: Calcium
CaMKII: Calmodulin-dependent protein kinase II
CARD: Caspase activation and recruitment domain
CASP8: Caspase 8
Cl^−^: Chloride
CLIC: Chloride intracellular channel protein
DAMPs: Damage-associated molecular patterns
DDX3X: Dead-box helicase 3 X-linked
DSB: Double-stranded breaks
ER: Endoplasmic reticulum
ETC: Electron transport chain
FADD: Fas-associated via death domain
GSDMD: Gasdermin
IL-18: Interleukin-18
IL-1: Interleukin-1
IL1R1: Interleukin-1 receptor, type 1
I_KUR_: Ultrarapid delayed rectifier potassium current
K^+^: Potassium
LDLR^−/−^: Low density lipoprotein receptor knockout
LPS: Lipopolysaccharide
LRR: Leucine-rich repeat
MARK4: Microtubule affinity-regulating kinase 4
MAVS: Mitochondrial antiviral signaling
mtDNA: Mitochondrial deoxyribonucleic acid
mtROS: Mitochondrial reactive oxygen species
Na^+^: Sodium
7: NEK Never in mitosis A (NIMA)-related kinase 7
NF-κB: Nuclear factor kappa-light-chain-enhancer of activated B cells
N-GSDMD: N-Gasdermin
NLRC4: NLR family CARD domain containing 4
NLRP1: NLR family pyrin domain containing 1
NLRP3: NOD-like receptor family pyrin domain-containing 3
NSAIDs: Non-steroidal anti-inflammatory drugs
P2X7R: P2X purinoceptor 7
PAMPs: Pathogen-associated molecular patterns
PML: Promyelocytic leukemia protein
PTMs: Post-translational modifications
PYD: Pyrin domain
RICVD: Radiation-induced cardiovascular diseases
RIPK1: Receptor-associated protein kinase-1
ROS: Reactive oxygen species
RyR2: Ryanodine receptor 2
RT: Radiation therapy
SIRT1: Sirtuin 1
SSB: Single-stranded breaks
T3SS: Type 3 secretion system
TGF-β: Transforming growth factor-beta
TLRs: Toll-like receptors
TNF: Tumor necrosis factor
TXNIP: Thioredoxin-interacting protein
VSMC: Vascular smooth muscle cells
